# Development and validation of the caregiver needs and resources assessment

**DOI:** 10.3389/fpsyg.2023.1063440

**Published:** 2023-03-17

**Authors:** Kin-Kit Li, Cyrus L. K. Leung, Dannii Yeung, Marcus Y. L. Chiu, Alice M. L. Chong, Beck C. Y. Lam, Edwin K. H. Chung, T. Wing Lo

**Affiliations:** ^1^Department of Social and Behavioural Sciences, City University of Hong Kong, Kowloon, Hong Kong SAR, China; ^2^The Jockey Club School of Public Health and Primary Care, The Chinese University of Hong Kong, Shatin, Hong Kong SAR, China; ^3^School of Health and Wellbeing, University of Bolton, Bolton, United Kingdom; ^4^Centre for Mental Health and Society, Bangor University, Bangor, United Kingdom; ^5^Felizberta Lo Padilla Tong School of Social Sciences, Caritas Institute of Higher Education, Tseung Kwan O, New Territories, Hong Kong SAR, China; ^6^Caritas Institute of Higher Education, Tseung Kwan O, New Territories, Hong Kong SAR, China

**Keywords:** caregivers, Hong Kong, assessment, scale development, caregivers’ needs, caregivers’ resources

## Abstract

**Introduction:**

Existing caregiver assessment tools were long criticized for focusing on the needs and burden while neglecting the importance of the resources. The current study aimed to develop a multidimensional and time-effective assessment tool that measures both needs and resources of non-paid family caregivers of older adults for screening and service-matching purposes.

**Methods:**

Items of the Caregiver Needs and Resources Assessment (CNRA) were developed from extensive literature reviews and focus group interviews of family caregivers and social workers in the field. In addition, we collected 317 valid responses from family caregivers of older adults from local non-government organizations in examining the psychometric properties of the CNRA.

**Results:**

The results revealed a 12-factor structure that fitted nicely into the conceptual frame of needs and resources domains. Need factors were positively associated with mental health symptoms, while resource factors were positively associated with peace in mind, meaning-making, and personal gain measures. The 36-item CNRA revealed good internal reliability and convergent validity.

**Discussion:**

The CNRA has the potential to be used as a compact yet balanced assessment tool for understanding both the needs and resources of caregivers for human service professionals.

## Introduction

The declining global fertility rate and extended longevity have significant implications for the healthcare systems and the support of family caregivers. In the year 2019’s global forecast, the number of people aged 65 years or over would be doubled from 703 billion in 2019 to 1.5 billion in 2050, and the proportion of older people would be up from one in 11 in 2019 to one in six people by 2050 (United Nations, Department of Economic and Social Affairs, and Population Division, 2020). Global aging goes with the increase in healthcare needs and costs of society, and a substantial growth conjecture is expected in the spending on long-term services and supports (Anastos-Wallen et al., 2020). Although caregiving by professionals is increasing in proportion to advancements in social and health services, a considerable amount of caregiving is still delivered by family members in high-, middle-, and low-income countries (Pandian et al., 2016; Hinton et al., 2019). Caregiving at home was deemed preferable by older adults for staying in their familiar living environment (Chui, 2008) and possibly reducing the probability of nursing home entry (Charles and Sevak, 2005). Family caregivers play the central position in the long-term care, and medical decision-making of their beloved family members with functional limitations in the self-care (Feinberg and Houser, 2012; Abreu et al., 2020), yet the demands for family caregivers of older family members are heavier than ever. Not only because the capacity to care for the older family members declines with the decreased household sizes and the increase in women’s employment rate, but our health system also relies more on family caregivers to provide care to older adults during crises such as the 2019 Coronavirus Disease (COVID-19) pandemic (Au Yeung et al., 2020; Kent et al., 2020). There is restricted access or curtailment in health and community services under the social distancing measures, which substantially disrupt the daily caregiving routine of the family caregivers (Lum et al., 2020; Chan et al., 2021), bring frustration to help-seeking (Kent et al., 2020), and impede access to medication (Wong and Cheung, 2020). Handling the household chores also brings fear of infection to the caregivers and the possible transmission to the care recipients (Kent et al., 2020; Lightfoot et al., 2021). Informal help availability and social support are reduced, making the family caregivers solely responsible for the caregiving tasks (Lightfoot et al., 2021; Savla et al., 2021). The increased use of online communication and activities may require family members’ assistance for older adults with low digital literacy (Kent et al., 2020). In the face of the growth of the aging population worldwide and the surge in caregiving demands amid the pandemic, we need a simple yet embracive triage tool in profiling the needs and resources of the caregivers to facilitate timely allocation of hit-the-spot support and interventions to the family caregivers in healthcare and community settings. Therefore, the aim of the project was to develop the Caregiver Needs and Resources Assessment (CNRA), a quick profiling tool that can help human service professionals, including social workers, medical and paramedical professionals, to understand caregivers’ situations and discuss the possible resources that can remediate their disrupted routines in the triage stage.

In the initial stage of case management, it is vital to distinguish between individuals who cannot cope with their current situation and thus assistance is urgently needed, and those who can overcome the caregiving burden on their own for more effective and efficient resource allocation (Jones and Griffiths, 2007). Being simple and easy are important attributes of an effective assessment tool for better acceptance and dissemination in self-administration and community setting (e.g., Chung et al., 2015). The development of CNRA was intended to be multi-faceted that enable the caregivers and human service professionals to get a quick initial idea about the unmet needs and resources possessed by the caregivers for further evaluation and referral of services. The human service professionals can, therefore, discuss with the caregivers for the services that best suit the caregivers’ situation among the available service options (Feinberg and Ellano, 2000; Kelly et al., 2013).

In the migration from a problem-solving and disease-based approach to strengths-based social work practice, the emphasis changes from remediating the immediate instrumental problems of the individuals to discovering and realizing the resources of the individuals from their environment as a resilient strategy during adversity (Guo and Tsui, 2010). An accurate understanding of the caregivers’ needs and resources is essential for a tailored intervention plan that can maximize their resources to reduce their burden, facilitate sustainable care for frail older adults, build their care capacity, and improve their quality of life.

One commonly used assessment tool among the non-government organizations (NGOs) that provide caregiver supporting services is the Zarit Burden Interview (ZBI; Zarit et al., 1980). This inventory has been adopted as an outcome measure in intervention studies (Tremont et al., 2013; e.g. Hashimoto et al., 2017) or in surveys (e.g., Cheng et al., 2013; Kwok et al., 2013) to evaluate the level of burden among caregivers. However, ZBI received critiques, including its “strain” focus and neglecting the positive impacts of caregiving (Chou et al., 2003) and the unstable and unreplicable factor structure in different caregiver samples (3-factor structure in Knight et al., 2000 and Springate and Tremont, 2014; 4-factor structure in Cheng et al., 2014; 5-factor structure in Lu et al., 2009). Other available assessment tools, for example, the Caregiver Self-Assessment Questionnaire (Epstein-Lubow et al., 2010), Behavioral and Instrumental Stressors in Dementia (Keady and Nolan, 1996), Comprehensive Needs Assessment Tool for Cancer Caregivers (Shin et al., 2011), the Caregiver Need Assessment (Moroni et al., 2008), and the Multidimensional Caregiver Burden Inventory (Novak and Guest, 1989) have been developed in the sake of measuring the needs of family caregivers in general or with care recipients of specific types of illness or limitations. Still, these tools fall short in capturing the resources possessed by the caregivers. This “pathological” view in assessing caregiver needs obscures the possibility of seeing how accepting the responsibility as the caregiver can facilitate the establishment of purpose, stimulation of personal growth, and cultivation of wisdom (Roth et al., 2015; Borson, 2021).

To capture the modifiable social contingencies potentially possessed by the caregivers that can shield the adversities in caregiving, the development of the CNRA was grounded on two dominant theoretical frameworks in the caregiving literature, namely, the Stress Process Model (SPM; Pearlin et al., 1990) and the Dual Process Model (DPM; Stroebe and Schut, 1999, 2001). SPM views caregiving stress as the product of the caregiving contexts, primary stressors (i.e., direct hardships anchored in caregiving), and secondary stressors (i.e., strains indirectly related to caregiving) that one is exposed to. Coping and resources, such as psychosocial maturity, social support, and better self-esteem (e.g., Benson, 2014), are mediators that can intervene in multiple points of the stress process, and the impact on caregivers vary as the mix of the stressors and resources vary considerably among caregivers. Under the tenet of SPM, caregiving stressors can be categorized into primary stressors stemming directly from the conditions and behaviors of the care recipients and secondary stressors generated by these conditions and behaviors. Two categories of secondary stressors are described: role strains (family conflict, job-caregiving conflict, economic problems, and construction of social life) and intrapsychic strains (self-esteem, mastery, loss of self, role captivity, competence, and gain). The SPM provides a valuable framework in identifying modifiable social factors in stressful encounters (Turner, 2009).

DPM describes bereavement as a dynamic process in the bereaved individual alternating between loss-orientation (dealing with the loss experience) and restoration-orientation (dealing with the arrangements for reorganizing one’s life without the deceased person). It suggests that a unique combination of stressors and resources, either contextual, social, or personal, would contribute to the varying appraisal of the stressful life event and its mental and physical health consequences. Although DPM pertains to the bereavement process, it echoes with SPM, emphasizing the interplay between stressors and resources in stressful life events under the stress and coping paradigm (Boerner et al., 2004).

Inspired by these models and given the inadequacy of the assessment tool in practice, we aimed to develop the Caregiver Needs and Resources Assessment (CNRA) which will facilitate social workers and healthcare professionals in the case management of caregivers such as triage, screening and service matching. In assessing its construct validity, we examined its relationship with common mental health and general well-being indicators, including caregiver quality of life, life satisfaction, and psychological distress.

## Methodology

### Item development and pilot test

In the initial phase of scale items development, we administered a semi-structured interview to four family caregivers and eight social workers from the four collaborating NGOs. The family caregivers interviewed were taking care of their frail older family members and were service users of the four collaborating NGOs. We asked questions tapped on their daily caregiving routines, major events of taking care of frail older adults, and their needs, resources, and coping strategies in caregiving in the semi-structured interview. Domains of needs and resources included psychological, physiological, social, environmental and others, and we openly asked about the sub-domains and other major domains that were relevant and important. We also consulted the social workers on the current practice of needs assessment in the interview. All interviews were audio-recorded with the participants’ consent.

Several themes emerged from the qualitative data and echoed with the literature regarding the needs and resources in caregiving. Interviewees brought up their psychological burden, including the sense of loss of self (Skaff and Pearlin, 1992; Noonan and Tennstedt, 1997), tiredness (Aarsland et al., 1999), the emergence of anxiety and depression symptoms (Pagel et al., 1985; Denno et al., 2013), and helplessness (Pagel et al., 1985). Various physiological symptoms were reported, including sleep problems, loss of appetite, and other health problems (van Wijngaarden et al., 2004; McCurry et al., 2007). Role conflicts with their family members, colleagues, and care recipients (Kramer and Kipnis, 1995; Stephens et al., 2001), and loss of social life (Skaff and Pearlin, 1992; Galvin et al., 2010), were common complaints among caregivers. Themes related to resources pertained spiritual and religious belief, coping strategies, meaning-making (Noonan and Tennstedt, 1997), resilience, moral commitment (Chang et al., 1998; Stuckey, 2001), the role recognition as a caregiver (Noonan and Tennstedt, 1997), support from professionals such as social workers, therapists (Reinhard, 1994; Sabella and Suchan, 2019) and community resources, support and recognition from other family members (Lidell, 2002), the closeness with the care recipient (Fauth et al., 2012; Rattinger et al., 2016), and healthy lifestyle (Mochari-Greenberger and Mosca, 2012).

Alongside these themes extracted in the qualitative analyses, we also extensively reviewed measurement tools in the field pertaining to the resources and needs of the caregivers, including the Multidimensional Caregiver Burden Inventory (Novak and Guest, 1989), Modified Caregiver Strain Index (Thornton and Travis, 2003), Work–Family Conflict Scale (Haslam et al., 2015), WHOQOL Spirituality, Religiousness and Personal Beliefs (SRPB) Field-Test Instrument (WHO, 2002), Perceived Social Support for Caregivers and Social Conflict scales (Goodman, 1991), and the measures assessing caregiving stress used in the study of Pearlin et al. (1990). We compiled an exhaustive list of caregiver needs and resources items. The research team has generated 133 questions based on the resources and needs dimensions. We presented the preliminary items to 14 family care and gerontology experts, including 10 social workers involved in caregiving support services and 4 researchers. They then commented on the provisional list of items regarding their relevance to the dimensions, uniqueness, and comprehensibility of the wordings. After the consultation, the provisional CNRA evolved into a 42-item version. We used a 5-point Likert scale (1 = *never*, 2 = *occasional*, 3 = *neutral*, 4 = *often*, 5 = *most of the time*) for the caregivers in evaluating their situation referring to the past month. We administered the 42-item version of CNRA to a group of 10 adult caregivers to collect their feedback regarding the potential problems in the administration, including the comprehensibility and phrasing of the items and the sequence of the items. We polished the items and kept the 42 items for the subsequent field test following the pilot test.

### Field testing

Ethical approval for human research was obtained from the Human Subject Ethics Sub-Committee of the City University of Hong Kong (Reference Number: 3-7-201905-01). Caregivers of frail older adults were recruited from four government-funded local community centers for older adults between August and December 2019. The four local community centers for older adults were located in the three main regions of Hong Kong (namely the Hong Kong Island, Kowloon, and the New Territories) and served needed caregivers of different socioeconomic statuses. Nonpaid caregivers who were taking care of a frail older adult (with at least one ADL or IADL difficulty) in their families and were aged 18 or above were eligible to participate. Paid formal caregivers were excluded from the study. Written consent was obtained from each participant at the beginning of the study, and the purpose and procedure of the study were explained. They were then asked to complete the survey. We interviewed illiterate participants with trained interviewers to complete the survey. The trained social workers interviewed the illiterate participants using the translated script provided by the research team to ensure that translated verbal Chinese in the script carried the equivalent semantic meaning as written Chinese on the questionnaire. Participation in this study was entirely voluntary, and a small financial compensation of HKD100 was provided to each participant after they had completed the questionnaire for their time and effort in participation. Service users of the participating NGOs were invited to join the study. We determined the target sample size based on the subject-to-variable ratios of 5:1 (Hatcher, 1994) to 10:1 (Nunnally, 1978), which required a sample size between 210 to 420 for our 42-item CNRA.

#### Procedures and measures

Considering the coverage of needs and resources of caregiving in the CNRA items, participants completed measures of caregiving burden, positive aspects of caregiving, general health status, personal gain, and peace of mind to test the construct validity of the scale. Considering our inclusion of both needs and resources related items in the pool, we expected the items to cluster into need-and resource-related factors. In examining the convergent validity of the CNRA, we used the extensively adopted caregiving burden scales, ZBI and MCBI, to verify the needs factors and the measurement of perceived resources for verifying the resource factors. We expected the need-related factors to be associated with general health and mental health measures in terms of predictive validity. In contrast, resource-related factors would be related to meaning-making and positive aspects of caregiving. Differences in the needs and resources factors were examined across demographic groups, including sex, age, relationship with the care recipients, and job status.

##### Caregiving burden

The perceived caregiving burden was assessed by the 12-item Chinese version of Zarit Burden Interview (ZBI; Tang et al., 2016) and the Multidimensional Caregiver Burden Inventory (MCBI; Novak and Guest, 1989). For ZBI, participants were asked to rate their agreement with each statement on a five-point Likert scale (1 = never, 5 = always). Sample items include “Do you feel that because of the time you spend with your relative that you do not have enough time for yourself?” and “Do you feel stressed between caring for your relative and trying to meet other responsibilities for your family or work?” A higher score indicates a higher perceived burden. The Cronbach’s alpha (α) of ZBI in our sample was 0.858.

The MCBI comprises 24 items covering five dimensions of burden, including time-dependence, developmental, physical, social, and emotional burdens. The perceived burden items were measured using a five-point Likert scale (1 = never; 5 = always). Sample items include “My care receiver needs my help to perform many daily tasks” and “I’m physically tired.” A higher score indicates a higher perceived burden. The Cronbach’s α of MCBI in our sample was 0.927.

##### Psychological distress

The caregivers’ psychological distress was assessed using the 12-item General Health Questionnaire (GHQ-12, Goldberg et al., 1997). Sample items of GHQ-12 include “Been losing confidence in self” and “Able to concentrate.” The participants rated the statement on a 5-point Likert scale ranging from 1 to 5 (instead of a 4-point Likert scale ranging from 0 to 3 as in the original GHQ-12 to maintain the consistency of scale point used in the survey). Higher GHQ-12 scores indicate more psychological distress. It had good reliability in our current sample (Cronbach’s α = 0.867).

##### General health status

The health status of the caregiver was evaluated by the Chinese version of the five-item of EuroQol-five dimensions (EQ-5D-5L; Cheung et al., 2016), covering five dimensions, namely, mobility, self-care, usual activities, pain and discomfort, and anxiety and depression. The participants were asked to rate their agreement with each statement using a five-point Likert scale (1 = no problem; 5 = severe problem) for all five items. To compute a single score in indicating the general health status of the caregivers, we adopted the method suggested by Devlin and Krabbe (2013) and Oppe et al. (2014), with scores of 1 and 0 indicating full health and death, respectively, and negative scores represent health status that is worse than death. We used the Hong Kong value set in computing the score (Wong et al., 2018), with the possible value ranging from 1 (for the health state 11,111) and-0.8637 (for the health state 55,555). The Cronbach’s α of ΕQ-5D-5L was 0.700.

##### Positive aspects of caregiving

The perceived positive aspects of caregiving were assessed by the Chinese version of the 11-item Positive Aspects of Caregiving Scale (PACS; Boerner et al., 2004; Lou et al., 2015). PACS (α = 0.924) comprises two factors, namely, enriching life (PACS_E; α = 0.894) and affirming self (PACS_A; α = 0.884). The participants were asked to rate their agreement on each statement on a five-point Likert scale (1 = strongly disagree; 5 = strongly agree). Samples items include “Feel strong and confident” and “Enable me to learn new skills.” A higher score of PACS indicates higher perceived benefits of engaging in caregiving.

##### Personal gain

The four-item Personal Gain Scale (PGS; Pearlin et al., 1990; Skaff and Pearlin, 1992) was employed to assess the caregiver’s personal growth and awareness of their inner strengths from the caregiving experience. The participants were asked to rate their agreement with each statement on a five-point Likert scale (1 = very much; 5 = not at all, instead of a four-point Likert scale in the original measure to keep the rating scale consistency in the survey) about inner strengths, self-confidence, growth, and new learning. Sample items include “Become more self-confident” and “Learned to do thing you did not do before.” A higher score represents more personal gain as a result of caregiving. The Cronbach’s α of PGS was 0.893.

##### Personal resources

We modified the 35-item Retirement Resources Inventory (RRI, Leung and Earl, 2012) to measure the perceived resources of caregivers. Participants rated the extent of each type of resource they had for assisting their caregiving tasks on a 5-point Likert scale from 1 to 5. Sample items of RRI included “I possess income to support my/my family living expenses” and “I would consider interactions with friends (in general) to be supportive.” A higher score represents higher perceived resources in the caregiver. The RRI revealed good reliability in our sample, with Cronbach’s α at 0.834.

##### Peace of mind

The perceived peace of mind of the caregiver was evaluated by the 7-item Peace of Mind scale (PoM; Lee et al., 2013; Yu et al., 2020). Participants were asked to rate their agreement with each statement on a 5-point Likert scale (1 = not at all; 5 = all the time). Sample items include “I feel content and comfortable with myself in daily life” and “My lifestyle gives me feelings of peace and stability.” The Cronbach’s α of PoM was 0.865.

#### Statistical analyses

A factor analysis was conducted using the principal axis factoring with oblimin rotation (as we expected that resulting factors would be correlated). A parallel analysis (Horn, 1965) was conducted on the 42 initial items by comparing the obtained eigenvalues from the factor analysis with the eigenvalues of a randomly generated dataset to determine the number of factors to be retained. Scree plot, Kaiser criterion (with an eigenvalue larger than 1.0), and the factor solution’s interpretability were also used as the aids of our judgment. The cutoff of the primary factor loading was set at 0.30 or above (Costello and Osborne, 2005), while the cutoff of the cross-loading was set at 0.30 or lower (Howard, 2016).

## Results

### Participants

We recruited 365 family caregivers of older adults. After discarding cases with incomplete responses of the CNRA, 317 participants were retained, with their mean age at 65.81 years (*SD* = 12.79, range = 27–95), and 79.18% being female. On average, they have been taking care of an older family member for 8.52 years (*SD* = 9.87, range = 0–65). Most of them were either spouse (45.89%) or child (46.52%) of the care recipients. Half of them (55.21%) have completed secondary or above education. There were 16.83% of the participants reported having a full-time job. Demographic information of the caregivers and care recipients are shown in Supplementary Tables 1, 2, respectively.

### Factor structure

The results of the parallel analysis suggested a factor solution of 12 factors, which explained 59.84% of the total variance (by calculating the mean of communalities). We dropped six items for their weak primary factor loading (less than 0.30) or strong cross-loading (greater than 0.30). The second round of parallel analysis and principal axis factoring was conducted on the remaining 36 items. Parallel analysis again suggested a 12-factor solution. The factor structure fit our data well (CFI = 0.912, AGFI = 0.945, RMSEA = 0.054, SRMR = 0.058). The factor loadings of the 36 items of CNRA are presented in Table 1. The 12 factors can be semantically divided into two dimensions, namely, needs and resources. The needs dimension included physiological needs (3 items), role conflict (3 items), care recipient’s needs (3 items), psychological needs (3 items), and social support needs (3 items). The resources dimension consisted of seven factors, labeled as spirituality (4 items), self-efficacy (3 items), responsibility and commitment as a caregiver (3 items), community resources (2 items), family support (3 items), healthy lifestyle (3 items), and closeness with care recipient (3 items). Table 2 presents the descriptive statistics and the correlations of the 12 factors of the CNRA. The CNRA subscales exhibited reliabilities, with Cronbach’s alpha ranging from 0.705 to 0.888.

**Table 1:** Factor loadings of the 36 items of the Caregiver Needs and Resources Assessment (*N* = 317).

	PHY	ROL	REP	PSY	SOC	SPI	RES	SEL	COM	FAM	CLO	HEA
1. Taking care of him/her deprives me of sleep.	0.505											
2. Taking care of him/her brings me poor health.	0.636											
3. Taking care of him/her brings me poor appetite.	0.474											
4. Taking care of him/her has impact on my job.		0.375										
5. Taking care of him/her brings me conflict with the family member.		0.574										
6. Taking care of him/her affect my family life.		0.581										
7. He/she has poor cognitive ability.			0.414									
8. His/her problematic behavior annoys me.			0.628									
9. He/she is emotionally disturbed.			0.560									
10. Taking care of him/her makes me confused.				0.610								
11. Taking care of him/her makes me lose interest in everything.				0.520								
12. I often have negative emotions (e.g., anxiety, sadness, frustration, annoyance).				0.481								
13. The responsibility of caregiving estranges me from my friends.					0.508							
14. I have less opportunities in keeping in touch with my friends.					0.926							
15. Taking care of him/her restricts my participation in social activities.					0.877							
16. My spiritual/ religious belief supports me in taking care of him/her.						0.471						
17. Taking care of him/her is meaningful.						0.344						
18. My faith/ religious belief helps me in understanding the difficulties in caregiving.						0.640						
19. Taking care of him/her helps me in understanding the meaning of life.						0.419						
20. I accept my identity as a family caregiver.							0.472					
21. No matter how hard it could be, I have the responsibility to take care of him/her.							0.705					
22. I am clear about the responsibilities as a caregiver.							0.397					
23. I know how to take care of him/her appropriately.								0.519				
24. I can effectively resolve the problems in caregiving.								0.829				
25. I can face and resolve the difficulties encountered during caregiving.								0.797				
26. I think the resources/services provided by my community for caregivers are satisfactory.									0.824			
27. I think the resources/services provided by my community for care recipients are satisfactory.									0.822			
28. My family can help me in tackling the difficulties in caregiving.										0.810		
29. I have good relationship with my family.										0.828		
30. When I encounter difficulties, I will at least have one family member from whom I can seek advice or support.										0.873		
31. He/she always makes me angry. (r)											0.868	
32. The trust between me and him/her declines. (r)											0.871	
33. I argue with him/her on trivial things.											0.486	
34. I can set a health plan and goal for myself.												0.669
35. I work out regularly.												0.702
36. I learn new skills or establish new habits.												0.632

PHY, physiological need; ROL, role conflict; REP, care recipient need; PSY, psychological need; SOC, social support need; SPI, spirituality; RES, responsibility; SEL, self-efficacy; COM, community resource; FAM, family support; CLO, closeness with care recipient; HEA, healthy lifestyle; (r), reversed item.

**Table 2:** Descriptive statistics of the 12 factors of the caregiver needs and resources assessment (*N* = 317).

	*M*	*SD*	α	PHY	ROL	REP	PSY	SOC	SPI	RES	SEL	COM	FAM	CLO	HEA
PHY	2.188	0.906	0.777	—											
ROL	1.983	0.937	0.711	0.542^***^	—										
REP	2.740	0.976	0.705	0.465^***^	0.511^***^	—									
PSY	2.161	0.940	0.778	0.624^***^	0.501^***^	0.515^***^	—								
SOC	2.556	1.143	0.888	0.581^***^	0.516^***^	0.485^***^	0.553^***^	—							
SPI	3.229	0.984	0.763	−0.042	0.165^**^	0.132	0.033	0.101	—						
RES	3.800	0.788	0.739	−0.020	0.092	0.106	−0.083	0.134^*^	0.556^***^	—					
SEL	3.287	0.835	0.828	0.054	0.149^**^	0.113^*^	0.040	0.133^*^	0.507^***^	0.531^***^	—				
COM	2.879	0.931	0.855	0.007	−0.016	−0.041	−0.026	−0.018	0.075	0.189^***^	0.204^***^	—			
FAM	2.970	1.263	0.883	−0.152^**^	−0.059	−0.082	−0.211^***^	−0.149^**^	0.163^**^	0.157^**^	0.050	−0.029	—		
CLO	3.326	0.882	0.825	−0.111^*^	−0.055	−0.051	−0.174^**^	−0.004	0.466^***^	0.494^***^	0.350^***^	0.161^**^	0.254^***^	—	
HEA	2.738	0.965	0.731	−0.106	−0.016	0.006	−0.115^*^	−0.175^**^	0.238^***^	0.127^*^	0.132^*^	0.105	0.111^*^	0.224^***^	—

PHY, physiological need; ROL, role conflict; REP, care recipient need; PSY, psychological need; SOC, social support need; SPI, spirituality; RES, responsibility; SEL, self-efficacy; COM, community resource; FAM, family support; CLO, closeness with care recipient; HEA, healthy lifestyle. ^*^p < 0.05, ^**^p < 0.01, ^***^p < 0.001.

### Convergent and predictive validity

The correlations between the CNRA factors and validation measures were shown in Table 3. The needs factors (i.e., physiological needs, role conflict, care recipient needs, psychological needs, and social needs) were positively correlated with caregiving burden (measured by ZBI, *r*s = 0.591–0.704, *p*s < 0.001; measured by MCBI, *rs* = 0.559–0.729, *ps* < 0.001), psychological distress (measured by GHQ, *rs* = 0.356–0.686, *ps* < 0.001), and negatively with general health status (measured by EQ5D, *rs* = −0.218 – -0.388, *ps* < 0.01). On the other hand, resources factors, including spirituality, self-efficacy, responsibility, community resources, family support, and healthy lifestyle, closeness with care recipients were found associating with peace of mind (*r*s = 0.185–0.381, *p*s < 0.001), affirming self (measured by PACS_A, *r*s = 0.122–0.372, *p*s < 0.05), enriching life (measured by PACS_E, *r*s = 0.155–0.536, *p*s < 0.01), positive gain (measured by PGS, *rs* = 0.146–0.498, *ps* < 0.01), and perceived resources (measured by RRI, *rs* = 0.117–0.416, *ps* < 0.05).

**Table 3:** Correlations between Caregiver Need Assessment constructs, burden and well-being measures (*N* = 317).

	*M*	*SD*	α	PHY	ROL	REP	PSY	SOC	SPI	RES	SEL	COM	FAM	CLO	HEA
GHQ (1–5)	2.590	0.612	0.867	0.507^***^	0.387^***^	0.377^***^	0.686^***^	0.356^***^	−0.108	−0.190^***^	−0.173^**^	−0.103	−0.218^***^	−0.294^***^	−0.187^***^
ZBI (1–5)	2.476	0.686	0.858	0.591^***^	0.617^***^	0.551^***^	0.704^***^	0.632^***^	0.144^*^	0.053	0.124^*^	−0.066	−0.192^***^	−0.005	−0.069
MCBI (1–5)	2.146	0.640	0.927	0.636^***^	0.623^***^	0.559^***^	0.729^***^	0.623^***^	0.052	0.042	0.122^*^	−0.047	−0.213^***^	−0.068	−0.082
EQ5D (−0.418–1)	0.815	0.197	0.700	−0.376^***^	−0.346^***^	−0.265^***^	−0.388^***^	−0.218^***^	0.042	0.020	−0.025	−0.003	0.113^*^	0.130^*^	0.147^**^
Peace (1–5)	3.353	0.738	0.865	−0.436^***^	−0.384^***^	−0.288^***^	−0.535^***^	−0.322^***^	0.190^***^	0.266^***^	0.191^***^	0.185^***^	0.190^***^	0.381^***^	0.324^***^
PACS_A (1–5)	2.938	0.902	0.884	0.009	−0.035	0.028	0.021	0.097	0.272^***^	0.371^***^	0.314^***^	0.138^*^	0.122^*^	0.372^***^	0.182^**^
PACS_E (1–5)	3.048	0.927	0.894	−0.054	−0.044	−0.039	−0.085	0.043	0.435^***^	0.442^***^	0.327^***^	0.166^**^	0.155^**^	0.536^***^	0.294^***^
PGS (1–5)	3.218	0.838	0.893	−0.063	−0.013	−0.008	−0.102	0.063	0.407^***^	0.396^***^	0.357^***^	0.146^**^	0.198^***^	0.498^***^	0.285^***^
RRI (1–5)	2.897	0.445	0.834	−0.191^***^	−0.125^*^	−0.089	−0.260^***^	−0.242^***^	0.343^***^	0.243^***^	0.242^***^	0.117^*^	0.233^***^	0.416^***^	0.348^***^

PHY, physiological need; ROL, role conflict; REP, care recipient need; PSY, psychological need; SOC, social support need; SPI, spirituality; RES, responsibility; SEL, self-efficacy; COM, community resource; FAM, family support; CLO, closeness with care recipient; HEA, healthy lifestyle. GHQ, General Health Questionnaire; ZBI, Zarit Burden Inventory; MCBI, Multidimensional Caregiver Burden Inventory; EQ5D, EuroQol-Five Dimensions; PACS, Positive Aspects of Caregiving Scale; PACS_A, Affirming self; PACS_E, Enriching life; PGS, Personal Gain Scale; RRI, Retirement Resources Inventory. ^*^p < 0.05, ^**^p < 0.01, ^***^p < 0.001.

### Demographic differences in caregiver’s needs and resources

There were demographic differences in caregivers’ needs and resources measured by the CNRA (Table 4). Gender differences were revealed in all of the need measures, and female caregivers reported higher needs in all domains than male caregivers. Caregivers of age 65 or above reported less role conflict, spiritual resources, and family support, but more community resources and a healthy lifestyle. Similarly, spouse caregivers reported less role conflict, spiritual resources, and family support, but higher physiological and psychological needs and more community resources. Caregivers who had full-time jobs reported less social support and a less healthy lifestyle.

**Table 4:** Mean differences in the caregivers’ needs and resources across demographic groups (*N* = 317).

	Male (*n* = 65)		Female (*n* = 251)			Below 65 (*n* = 137)		65 or above (*n* = 174)			Not spouse (*n* = 171)		Spouse (*n* = 145)			Without fulltime job (*n* = 262)		With fulltime job (*n* = 53)		
	*M*	*SD*	*M*	*SD*	*t*	*M*	*SD*	*M*	*SD*	*t*	*M*	*SD*	*M*	*SD*	*t*	*M*	*SD*	*M*	*SD*	*t*
PHY	1.79	(0.71)	2.30	(0.92)	−4.18^***^	2.14	(0.82)	2.24	(0.97)	−0.95	2.08	(0.82)	2.32	(0.98)	−2.30^*^	2.21	(0.93)	2.08	(0.73)	0.93
ROL	1.75	(0.86)	2.05	(0.95)	−2.26^*^	2.12	(0.89)	1.87	(0.86)	2.41^*^	2.10	(0.89)	1.86	(0.98)	2.29^*^	1.98	(0.97)	2.04	(0.77)	−0.48
REP	2.51	(0.98)	2.81	(0.97)	−2.23^*^	2.76	(0.91)	2.75	(1.03)	0.15	2.74	(0.93)	2.76	(1.03)	−0.19	2.74	(0.98)	2.79	(0.93)	−0.37
PSY	1.75	(0.72)	2.27	(0.96)	−4.09^***^	2.12	(0.87)	2.19	(1.01)	−0.65	2.02	(0.85)	2.33	(1.02)	−2.89^**^	2.18	(0.97)	2.09	(0.80)	0.65
SOC	2.26	(1.05)	2.64	(1.15)	−2.39^*^	2.55	(1.12)	2.57	(1.17)	−0.14	2.44	(1.11)	2.69	(1.17)	−1.92	2.62	(1.17)	2.26	(0.94)	2.09^*^
SPI	3.09	(0.95)	3.27	(0.99)	−1.29	3.47	(0.93)	3.04	(0.98)	3.93^***^	3.39	(0.96)	3.06	(0.98)	3.02^**^	3.19	(0.99)	3.44	(0.93)	−1.70
RES	3.76	(0.75)	3.82	(0.79)	−0.53	3.83	(0.83)	3.78	(0.76)	0.62	3.80	(0.82)	3.82	(0.74)	−0.26	3.81	(0.78)	3.77	(0.81)	0.33
SEL	3.25	(0.84)	3.30	(0.84)	−0.41	3.30	(0.79)	3.27	(0.88)	0.33	3.27	(0.81)	3.31	(0.87)	−0.40	3.33	(0.83)	3.13	(0.79)	1.63
COM	2.94	(0.88)	2.87	(0.95)	0.57	2.73	(0.89)	2.99	(0.96)	−2.45^*^	2.76	(0.87)	3.02	(0.98)	−2.52^*^	2.93	(0.93)	2.67	(0.91)	1.88
FAM	3.02	(1.19)	2.96	(1.28)	0.32	3.20	(1.26)	2.83	(1.24)	2.61^**^	3.12	(1.23)	2.80	(1.29)	2.25^*^	2.92	(1.25)	3.20	(1.33)	−1.51
CLO	3.42	(0.76)	3.31	(0.91)	0.93	3.40	(0.87)	3.27	(0.90)	1.31	3.37	(0.86)	3.27	(0.91)	1.06	3.31	(0.88)	3.38	(0.91)	−0.53
HEA	2.79	(0.86)	2.73	(0.99)	0.43	2.61	(0.89)	2.83	(1.02)	−1.98^*^	2.72	(0.92)	2.77	(1.02)	−0.50	2.80	(0.97)	2.47	(0.91)	2.28^*^

PHY, physiological need; ROL, role conflict; REP, care recipient need; PSY, psychological need; SOC, social support need; SPI, spirituality; RES, responsibility; SEL, self-efficacy; COM, community resource; FAM, family support; CLO, closeness with care recipient; HEA, healthy lifestyle. ^*^p < 0.05, ^**^p < 0.01, ^***^p < 0.001.

## Discussion

The current study developed and examined the psychometric properties of a caregiver needs and resources assessment, an indigenously developed screening tool tapping the needs and resources of caregivers who provide support and care to their older adult family members. In our field testing, the exploratory factor analysis revealed a 12-factor model for the 36 items retained in the CNRA. The 12 factors can be semantically categorized into needs factors and resources factors. In examining the convergent validity, all needs factors positively correlated with more pathological or negative constructs, including mental health symptoms and caregiving burden, and negatively correlated with general health. All resources factors are positively associated with positive constructs, including meaning-making, peace of mind, and perceived resources.

The five needs factors tapped into the stressors or burden in different domains, including the burden on the caregivers’ health (physiological needs; e.g., Vitaliano et al., 2003), deprivation of social life (social needs; e.g., Palamaro Munsell et al., 2012), psychological burden (psychological needs; e.g., Baillie et al., 1988; González-Salvador et al., 1999), conflicts between different roles (role conflict; e.g., Kramer and Kipnis, 1995; Gordon et al., 2012), and the functional deterioration of the care recipients (Ornstein and Gaugler, 2012; care recipient needs; e.g., Lin, 2020). These factors reflect the stressors of the caregivers on different facets that compose the needs to be fulfilled or addressed, which is often the main focus of the caregiver assessment tools in the field (e.g., ZBI and MCBI). The association between caregiving burden and mental and physical health was well discussed in the literature (e.g., Chang et al., 2010; de Oliveira et al., 2015; Del-Pino-Casado et al., 2019), especially when the care recipients are in poor functional capacity (e.g., Morimoto, 2003). Our five factors on the needs dimension have revealed good convergent validity for their positive associations with ZBI and MCBI. These need factors also revealed good predictive validity for positive associations with mental health symptoms and negative associations with general health status.

As advocated in SPM and DPM, integrated consideration of both the needs or burden experienced and the resources possessed by the caregivers are needed to understand the caregivers’ appraisal of their stressful events (Pearlin et al., 1990; Stroebe and Schut, 1999). More studies stress the importance of considering the positive aspects and resources in the caregiving (Kramer and Kipnis, 1995; Boerner et al., 2004; Yu et al., 2018). Preserving and enhancing the positive aspects and resources of the caregivers can serve to provide protective factors to the caregivers in the face of stress (Yu et al., 2018). It is also suggested that the positive aspects and resources during the caregiving process could enhance the adaptation after the loss of care recipients (Boerner et al., 2004).

In our CNRA, we have seven factors related to the resources domain, including the spirituality and religious belief that provide meaning and substance in life and the way to handle loss and suffering (spirituality; e.g., Chang et al., 1998; Puchalski and Sandoval, 2003; Puchalski, 2012), self-efficacy or perceived capacity in carrying out caregiving tasks (self-efficacy; e.g., Merluzzi et al., 2011; Cheng et al., 2014; Durmaz and Okanlı, 2014; Yildiz et al., 2017), the commitment and perceived responsibility in taking care of the their frail family members (responsibility; e.g., Häggström et al., 2010; Qiu et al., 2018), community services and resources (community resources; e.g., Yamashita and Amagai, 2008; Weber et al., 2011), the instrumental and emotional support from other family members (family support; e.g., Lidell, 2002; Fauth et al., 2012; Feng and Magen, 2016), the awareness in maintaining good health and wellness (healthy lifestyle; e.g., Beesley et al., 2011), as well as the emotional closeness with the care recipients (closeness with care recipients; e.g., Lyonette and Yardley, 2003). The resources factors revealed good convergent validity in their positive associations with RRI and PACS. They also showed good predictive validity in positively associating with peace in mind and perceived gain.

The demographic analyses revealed important caregivers’ needs and resources between demographic groups. Specifically, male caregivers reported fewer needs than female caregivers. This evidence again supported the notion of the gendered nature of caregiving (Xiong et al., 2020). For example, in comparison with female caregivers, male caregivers are less likely to consider it a family or filial obligation to take care of their frail family members As such, they are more willing to seek help, share the caregiving burden with others, and engage in activities that provide respite from the caregiving burden (Brown and Chen, 2008; Xiong et al., 2020). Interestingly, younger and non-spouse (who were usually the child) caregivers reported more role conflicts, more spiritual resources, more family support but fewer community resources. In terms of resources, younger caregivers are higher in digital literacy and more accessible to online resources. This is particularly important during the COVID-19 pandemic to look for spiritual resources and connect with the family members online. In Hong Kong, since resources for caregivers are offered mainly through the services of older people in social services organizations, the services available may not fit the needs of younger and working caregivers or be able to reach them. Caregivers with full-time jobs reported less social support needs and a less healthy lifestyle. A full-time job could provide social support from the colleagues and supervisors (Boumans and Dorant, 2021). Yet, dual responsibilities dealing with the job and caregiving tasks may bring a less healthy lifestyle (Bainbridge et al., 2021).

To our best knowledge, CNRA is the first assessment tool that provides a one-stop solution in assessing and differentiating caregiver needs and resources with good psychometric properties. We believe the development of the CNRA would have several important practical implications. First, the extensive coverage of caregiving needs and resources would be useful in a case management setting as a quick triage tool to overview the profile of needs and resources of a particular caregiver. It could serve as a reminder for both the case managers and the clients not to overlook a balanced view of both needs and resources that the clients carry. Second, the CNRA can facilitate the prioritization and tailor-making of the services and intervention plans that best suit the situation of a particular caregiver. With the easily interpretable scores of CNRA on hand, the case managers can discuss with clients their needs and resources in working on the preferred case management plans and objectives. Third, this scale can be applied in the social work training module for caregiver needs and resources assessment, as there is no standardized assessment tool in the field. Using the CRNA makes it possible to unify the assessment quality for novice and experienced social workers. CNRA also offers an embracive profile of caregiving needs and resources that takes around an hour or less for the initial triage interview. Timely and accurate assessment of the needs and resources of caregivers can make quick allocation of services and development of intervention plans possible and, in turn, lower their caregiving burden as quickly as possible. Theoretically, CNRA could serve as a handy measurement tool in verifying the relationship between caregivers’ needs and resources in future studies.

We admitted there were several limitations in the current findings. First, because the sample was recruited from the caregivers who were using services in the collaborating NGOs in Hong Kong, further studies would be needed in other samples of caregivers who are not using NGOs services, as well as samples from different cultures, to examine the stability of the factor structure revealed in the current study, Second, the validation of the CNRA relied on self-report measures. The use of more objective outcome measures can better verify the scale’s construct validity. Third, the cross-sectional design did not enable us to draw a strong conclusion about the predictive validity of the factors. The use of longitudinal design would be helpful to see if the needs and resources factors can predict mental health symptoms and meaning-making in the caregiving process at a later time in future studies. Despite the limitations, we believe the CNRA would be a helpful tool for the case management of caregivers in different service settings.

The development of the CNRA serves to respond to the paradigm shift from a disease-based and problem-solving approach to a strength-and resilience-based approach to managing caregiving stress. The CNRA, as a triage tool, provides a balanced and embracive view of both needs and resources in handling caregiving stress, which would benefit both the clients and workers in liaising the development of a tailored-made intervention plan.

## Author’s note

The Chinese version of CNRA was validated in this study. The English version presented in the article was for demonstration only. The Chinese items and the scoring system are presented in Supplementary Table 3. This scale can be used for non-commercial research and educational purposes without seeking written permission.

## Data availability statement

The raw data supporting the conclusions of this article will be made available by the authors, without undue reservation.

## Ethics statement

The studies involving human participants were reviewed and approved by Human Subject Ethics Sub-Committee of City University of Hong Kong (Reference Number: 3-7-201905-01). The patients/participants provided their written informed consent to participate in this study.

## Author contributions

K-KL, DY, MC, and AC conceptualized the study, developed the methodology, and obtained funding support. BL managed data collection. K-KL, CL, EC, and BL analyzed and interpreted the data. TWL supervised the execution of the project. CL wrote the manuscript. All authors contributed to the article and approved the submitted version.

## Funding

This work was supported by the Simon K.Y. Lee Foundation.

## Conflict of interest

The authors declare that the research was conducted in the absence of any commercial or financial relationships that could be construed as a potential conflict of interest.

## Publisher’s note

All claims expressed in this article are solely those of the authors and do not necessarily represent those of their affiliated organizations, or those of the publisher, the editors and the reviewers. Any product that may be evaluated in this article, or claim that may be made by its manufacturer, is not guaranteed or endorsed by the publisher.
