# Supplementary material for: Development and validation of the caregiver needs and resources assessment
Source: Front Psychol. 2023 Mar 17;14:1063440. doi: 10.3389/fpsyg.2023.1063440 (PMC10064064; doi:10.3389/fpsyg.2023.1063440)
Supplement: Supplementary file 1 [file Table_1.pdf]

Supplementary Table 1. Descriptive statistics of the demographics of the caregivers

	Mean/% (Range)	SD
Age	65.81(27 – 95)	12.79
25-44	3.86%	
45-64	40.19%	
65-84	48.88%	
85+	7.07%	
Biological sex of the caregivers		
Female	79.18%	
Years of taking care of an older family member	8.52 (0 – 65)	9.87
0-5 years	53.21%	
6-10 years	24.04%	
11-20 years	15.06%	
20 years +	7.69%	
Monthly household income	HKD15123	HKD15044
Below HKD 5000	12.95%	
HKD 50001-10000	41.73%	
HKD 10001-30000	36.69%	
HKD 30001-50000	6.12%	
HKD 50001or above	2.52%	
Relationship with care recipients		
Spouse	45.89%	
Child	46.52%	
Others (e.g., siblings, other relatives)	7.59%	
Having full-time job	16.72%	
With religious belief	61.20%	
Married and living together with spouse	61.83%	
With chronic illness(es)	78.55%	
With high school or higher education level	55.21%	
Income sources		
Salary	26.50%	
Monetary support from family	48.58%	
Saving/ investment	42.27%	
Old Age Allowance	28.71%	
Disability Allowance	6.31%	
Old Age Living Allowance	31.23%	
Comprehensive Social Security Assistance	8.28%	
