# Supplementary material for: Development and validation of the caregiver needs and resources assessment
Source: Front Psychol. 2023 Mar 17;14:1063440. doi: 10.3389/fpsyg.2023.1063440 (PMC10064064; doi:10.3389/fpsyg.2023.1063440)
Supplement: Supplementary file 2 [file Table_2.pdf]

Supplementary Table 2. Descriptive statistics of the demographics of the care recipients

	Mean/% (Range)	SD
Age	83.44 (57-109)	8.47
55-64	1.91%	
65-74	13.97%	
75-84	32.38%	
85+	51.75%	
Biological sex of the care recipients		
Female	53.32%	
With chronic illness(es) (care recipients)	99.69%	
