# Supplementary material for: Development and validation of the caregiver needs and resources assessment
Source: Front Psychol. 2023 Mar 17;14:1063440. doi: 10.3389/fpsyg.2023.1063440 (PMC10064064; doi:10.3389/fpsyg.2023.1063440)
Supplement: Supplementary file 3 [file Table_3.pdf]

Supplementary Table 3. Chinese version of the Caregiver Needs and Resources Assessment

在過去一個月照顧被照顧者時，你多常有以下的想法？請圈選相應的數字。

1＝從不；2＝有些；3＝一般；4＝經多；5＝極多

1. 照顧他／她令我缺乏足夠的睡眠
2. 照顧他／她令我健康變差
3. 照顧他／她以來，我胃口變差
4. 照顧他／她影響了我的工作
5. 照顧他／她令我和其他家人有意見分歧
6. 照顧他／她影響了我自己的家庭生活
7. 他／她的認知能力差
8. 他／她的問題行為令我感到困擾
9. 他／她的情緒起伏不定
10. 照顧他／她令我感到不知所措
11. 照顧他／她令我對事情失去興趣
12. 我經常感受到負面的情緒（例如：焦慮、悲傷、苦惱、煩躁等）？
13. 照顧的責任令我和朋友的關係變得疏離
14. 我和朋友接觸的機會減少了
15. 照顧他／她令我減少參與社交活動
16. 我的信念／信仰支持我繼續照顧他／她
17. 照顧他／她是有意義的
18. 我的信念／信仰幫助我理解照顧的艱難
19. 照顧他／她令我明白生命的意義
20. 我接納護老者的身份
21. 無論感到多辛苦，我也有責任去照顧他／她
22. 我清楚我照顧者身份所肩負的責任
23. 我懂得如何妥善照顧他／她
24. 我能有效地解決照顧上遇到的問題
25. 我能面對逆境，解決照顧上的困難
26. 我認為現時社區中提供的護老者服務能滿足我的需要
27. 我認為現時社區中提供的被照顧者服務／配套能滿足被照顧者的需要
28. 家人幫助我應對照顧的困難
29. 我與家人的關係良好
30. 遇到困難時，至少有一個信任的家人可以為我提供協助／建議
31. 他／她經常惹我生氣(r)
32. 我和他／她之間的信任減少了(r)
33. 我和他／她為小事爭執
34. 我能為自己訂立健康計劃和目標
35. 我定期做運動
36. 我學習一些新事物／建立新興趣

Notes. (r) = reversed item.
